# Protection of the marginal mandibular branches of the facial nerves by different surgical procedures in comprehensive cervical lymphadenectomy for locally advanced oral and oropharyngeal cancer: a multicenter experience

**DOI:** 10.1186/s12957-023-02913-1

**Published:** 2023-01-31

**Authors:** Jiuzhou Zhao, Wen Zeng, Chengyu Qiu, Jiafeng Liu, Ke Li, Jing Huang, Michael C. F. Tong, Xiangmin Zhang

**Affiliations:** 1 Department of Otolaryngology, Longgang E.N.T Hospital & Shenzhen Key Laboratory of E.N.T, Institute of E.N.T Shenzhen, Guangdong Province, No.3004, Longgang Avenue, Shenzhen, People’s Republic of China; 2 Department of Head and Neck, Tumor Hospital of Ganzhou, Ganzhou, Jiangxi Province People’s Republic of China; 3 Department of Oral and Maxillofacial Surgery, First Hospital of Qiqihaer City, Heilongjiang Province, Qiqihaer, People’s Republic of China; 4 Department of Oral and Maxillofacial Surgery, First Affiliated Hospital of Gannan Medical University, Ganzhou, Jiangxi Province People’s Republic of China; 5 Department of Institute of Cancer Research, Tumor Hospital of Ganzhou, Ganzhou, Jiangxi Province People’s Republic of China; 6 Department of Otorhinolaryngology, Head & Neck Surgery, The Chinese University of Hong Kong, Shatin, New Territories, Hong Kong SAR, People’s Republic of China

**Keywords:** Head and neck surgery, Cervical lymphadenectomy, Marginal mandibular branches of facial nerves, Surgical approach

## Abstract

**Objective:**

According to the different characteristics of patients and cervical lymph node metastasis of oral and oropharyngeal cancer, the marginal mandibular branches of facial nerves were treated by different surgical procedures, and the safety and protective effects of different surgical procedures were investigated.

**Methods:**

One hundred ninety-seven patients with oral and oropharyngeal cancer satisfying the inclusion criteria were selected. According to the different characteristics of patients and cervical metastatic lymph nodes, three different surgical procedures were used to treat the marginal mandibular branches of the facial nerve: finding and exposing the marginal mandibular branches of the facial nerves at the mandibular angles of the platysma flaps, finding and exposing the marginal mandibular branches of facial nerves at the intersections of the distal ends of facial arteries and veins with the mandible, and not exposing the marginal mandibular branches of the facial nerves. The anatomical position, injury, and complications of the marginal mandibular branches of the facial nerves were observed.

**Results:**

The marginal mandibular branches of the facial nerves were found and exposed at the mandibular angles of the platysma flaps in 102 patients; the marginal mandibular branches of facial nerves were found and exposed at the intersections of the distal ends of the facial arteries and veins with the mandibles in 64 patients; the marginal mandibular branches of facial nerves were not exposed in 31 patients; among them, four patients had permanent injury of the marginal mandibular branches of the facial nerves, and temporary injury occurred in seven patients. There were statistically significant differences in the protection of the mandibular marginal branch of the facial nerve among the three different surgical methods (*P* = 0.0184). The best protective effect was to find and expose the mandibular marginal branch of the facial nerve at the mandibular angle of the platysma muscle flap, and the injury rate was only 2.94%.

**Conclusion:**

The three different surgical procedures were all safe and effective in treating the marginal mandibular branches of the facial nerves, the best protective effect was to find and expose the mandibular marginal branch of the facial nerve at the mandibular angle of the platysma muscle flap.

## Introduction

Comprehensive cervical lymphadenectomy includes all the lymphoid and adipose tissues that can be dissected in classical radical cervical lymphadenectomy, and whether the internal jugular veins, sternocleidomastoid muscles, and accessory nerves are preserved does not affect whether it is classified as comprehensive [1]. Radical resection or radical resection plus repair and reconstruction with comprehensive cervical lymphadenectomy is the main method for comprehensive treatment of locally advanced oral and oropharyngeal cancer, although there is evidence that the submandibular glands can be spared in cervical lymphadenectomy for early-stage oral and oropharyngeal cancer [2–5]. However, removal of the submandibular glands is inevitable in cervical lymphadenectomy for locally advanced oral and oropharyngeal cancer [6–8].

Surgeries involving the facial nerves have a high risk of iatrogenic injuries. The marginal mandibular branches of the facial nerves are extremely special in nature. Once an injury occurs, the complications will be devastating and functions will be lost, which will involve the patient’s facial aesthetics [9–11], causing great psychological trauma to the patient, and thus affecting tumor treatment [12, 13]. Whether it is necessary to dissect the marginal mandibular branches of the facial nerves when removing the submandibular glands during comprehensive cervical lymphadenectomy and whether the dissection can protect the marginal mandibular branches of the facial nerves are a subject worthy of clinical research. In this study, according to the different characteristics of patients and cervical lymph node metastasis of oral and oropharyngeal cancer, different surgical procedures were used to treat the marginal mandibular branches of the facial nerves, and the safety and protective effects of different surgical procedures were investigated, so as to further summarize the clinical experience, reduce the risk of iatrogenic injury to the marginal mandibular branches of the facial nerves, and maintain the aesthetics and functions of patients’ faces to the maximum extent.

## Materials and methods

### Ethics statement

The Ethical Committee of the Longgang E.N.T Hospital approved clinical samples for research purposes (NO. 2022–0001), and this study conformed to the principles contained in the World Medical Association Declaration of Helsinki. Informed consent was requested as anonymous specimens and was given by all human participants in this study.

### Case inclusion and exclusion criteria

The inclusion criteria include ① age > 18 years; ② definitive pathological diagnosis of oral and oropharyngeal cancer requiring cervical lymphadenectomy with submandibular gland resection according to the National Comprehensive Cancer Network guidelines or the results of multidisciplinary care discussion; ③ no significant invasion of the marginal mandibular branches of the facial nerves before operation; ④ no serious cardiovascular diseases, diabetes, chronic respiratory diseases, cerebrovascular diseases, etc.; ⑤Karnofsky score ≥ 80 points; ⑥ an estimated survival of more than one year; ⑦ and patients who voluntarily participated and signed the informed consent form.

The exclusion criteria include ① cervical lymphadenectomy for oral and oropharyngeal cancer without the need for submandibular gland resection, ② significant invasion of the marginal mandibular branches of the facial nerves before operation, ③ significant submandibular lesions, ④ a history of surgery or radiotherapy of the neck, ⑤ distant metastasis, and ⑥ unable to tolerate the surgery due to severe comorbidities.

### Clinical data

**Fig. 1:**
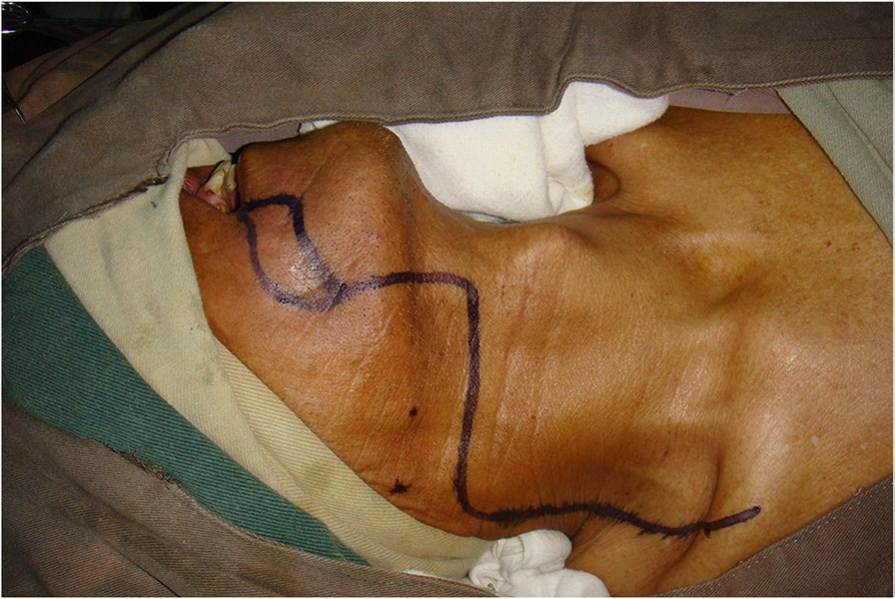
Incision of the buccal mucosa cancer invading the skin

**Fig. 2:**
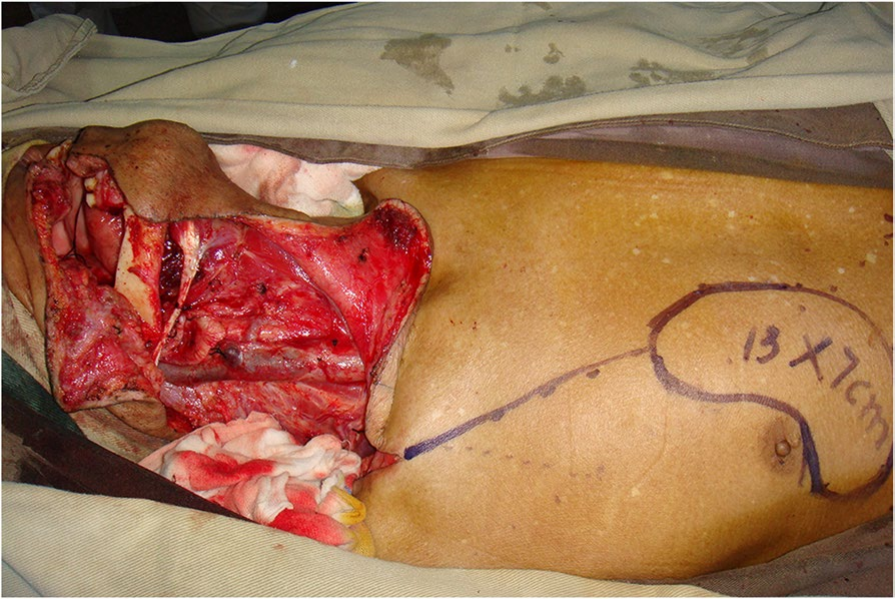
Comprehensive cervical lymphadenectomy with dissection of the marginal mandibular branches of the facial nerves plus extended radical resection of the right buccal cancer

**Fig. 3:**
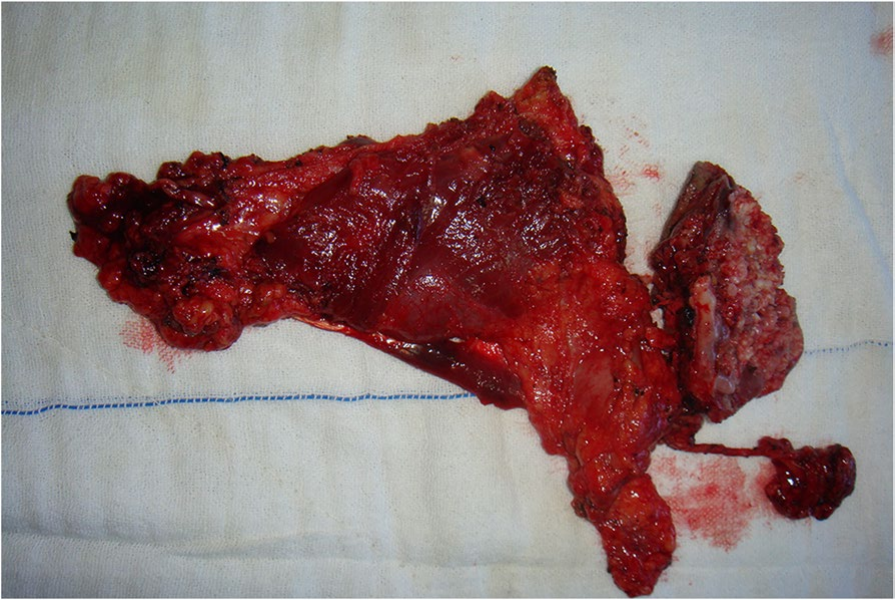
En bloc resection specimen

**Fig. 4:**
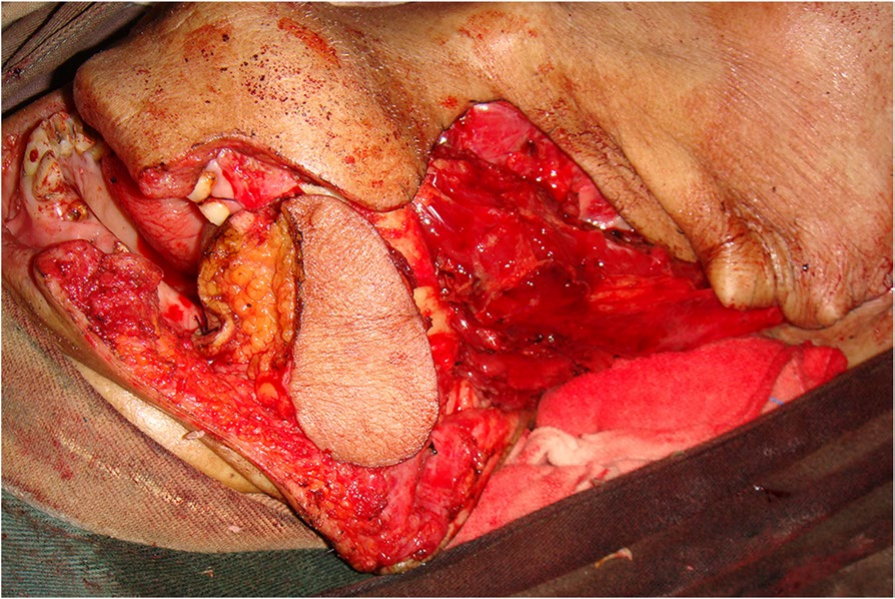
Repair of penetrating buccal defect with a pectoralis major flap folded in half

**Fig. 5:**
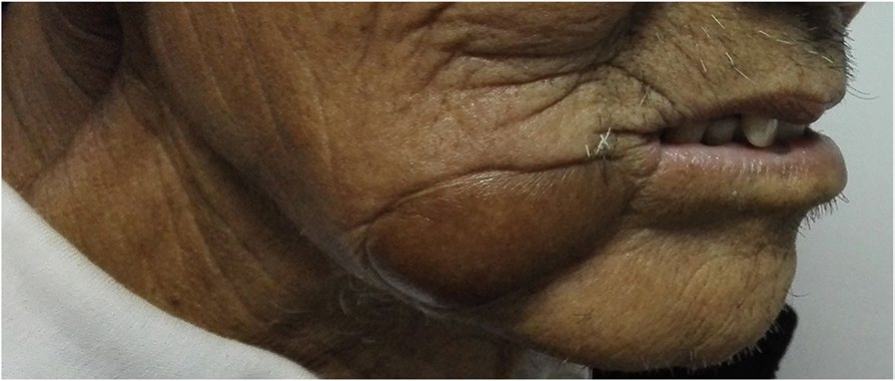
Re-examination at 3 years after surgery showed that the patient had a good appearance and the marginal mandibular branches of the facial nerves were functioning normally

**Fig. 6:**
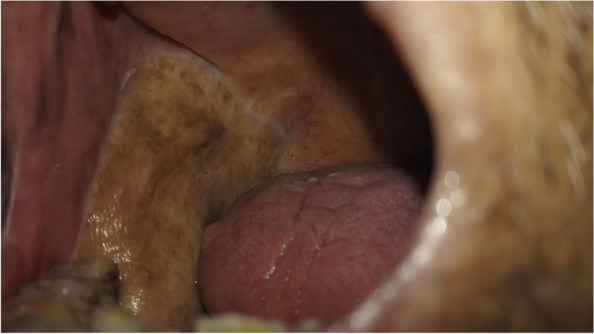
Appearance of the intraoral flap repair 3 years after surgery

From January 2014 to June 2021, 197 patients with oral and oropharyngeal cancer, including 131 males and 66 females, aged 29 to 68 years, and with a median age of 54.6 years, were selected from the Head and Neck Department of Shenzhen Otolaryngology Research Institute/Shenzhen Longgang Otolaryngology Hospital, Head and Neck Department of Gannan Medical University Affiliated Cancer Hospital, Department of Oral and Maxillofacial Surgery of the First Hospital of Qiqihar in Heilongjiang Province, and Department of Otorhinolaryngology-Head and Neck Surgery of First Affiliated Hospital of Gannan Medical University. There were 78 cases of tongue cancer, 35 cases of gingival cancer, 32 cases of buccal cancer, 28 cases of oral floor cancer, 24 cases of lingual root or oropharyngeal cancer, 16 cases at stage III, 114 cases at stage IVA, and 67 cases at stage IVB (Table 1). All patients underwent radical tumor resection or radical tumor resection plus flap repair and reconstruction with cervical lymphadenectomy and submandibular gland resection. The marginal mandibular branches of the facial nerves were treated and submandibular glands were removed during the operation (Figs. 1, 2, 3, 4, 5, and 6).

**Table 1:** General clinical data of the patients (*n* = 197)

Parameter	Number of patients, *n* (%)
Age, years	
25 ~ 39	10 (5.08)
40 ~ 49	33 (16.75)
50 ~ 59	87 (44.16)
60 ~ 69	67 (34.01)
Sex	
Male	131(66.50)
Female	66 (33.50)
KPS	
80 ~ 89	41 (20.81)
≥ 90	156(79.19)
Type of tumor	
Tongue cancer	78 (39.59)
Gingival carcinoma	35 (17.77)
Buccal cancer	32 (16.25)
Carcinoma of the mouth floor	28 (14.21)
Cancer of the tongue or oropharynx	24 (12.18)
TNM stages	
III	16 (8.12)
IVA	114(57.87)
IVB	67 (34.01)
Postoperative treatment	
Radiotherapy	108(54.82)
Concurrent radiotherapy	89 (45.18)

*KPS* Karnofsky performance score

### Evaluation criteria for facial nerve function

The facial nerve function was evaluated using the Sunnybrook [14] facial nerve evaluation system. According to this scoring criteria, the higher the score, the better the facial nerve function. The facial nerve function is normal if the scores before and after surgery are the same, and the score after surgery being lower than that before surgery indicates facial nerve dysfunction. In this study, the function of the marginal mandibular branches of the facial nerves was mainly evaluated.

### Surgical approaches

All operations were performed by doctors with the title of an associate chief physician or above and with several years of surgical experience. The most suitable surgical incision was selected according to the characteristics of oral and oropharyngeal cancer and the results of the multidisciplinary care discussion. According to the different characteristics of patients and cervical metastatic lymph nodes, three different surgical procedures were used to treat the marginal mandibular branches of the facial nerves: finding and exposing the marginal mandibular branches of the facial nerves at the mandibular angles of the platysma flaps, finding and exposing the marginal mandibular branches of the facial nerves at the intersections of the distal ends of facial arteries and veins with the mandible, and not exposing the marginal mandibular branches of the facial nerves.

**Fig. 7:**
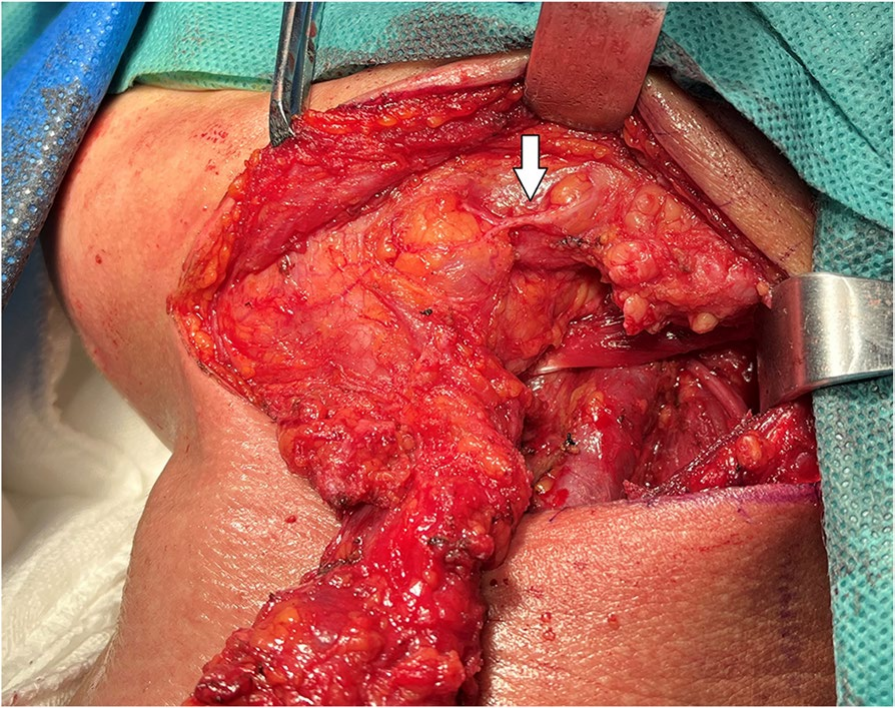
Searching for the marginal mandibular branch of the facial nerve at the mandibular angle

**Fig. 8:**
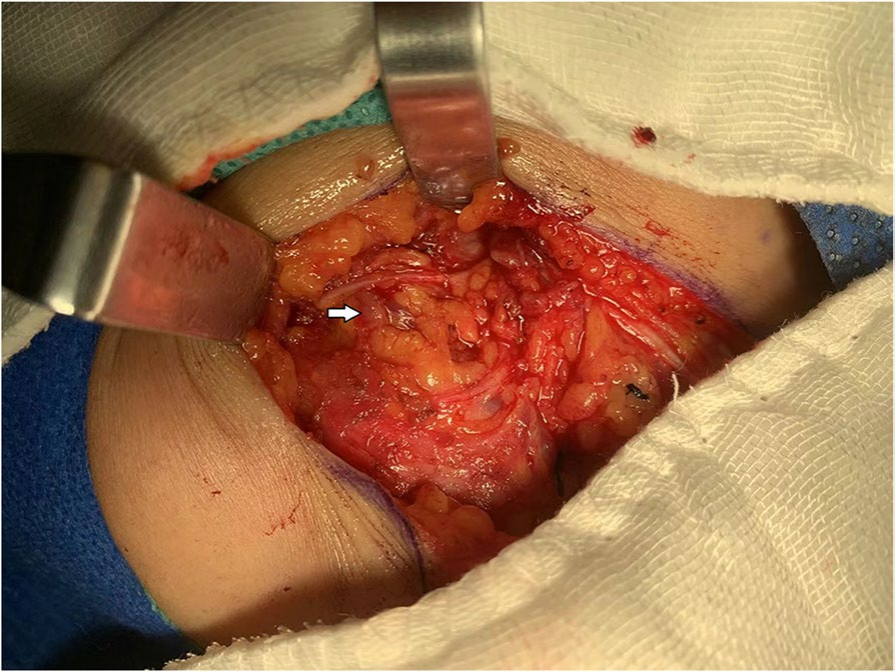
Searching for the marginal mandibular branch of the facial nerve at the distal ends of the facial artery and vein

Finding and exposing the marginal mandibular branches of the facial nerves at the mandibular angles of the platysma flaps: At the junction of about 1 cm superior to the mandibular angle and 1 cm anterior to the ascending branch of the mandible, an electric scalpel was used to make an incision along the horizontal plane of the mandible, and then, the adipose tissues were carefully cut open, the marginal mandibular branch of the facial nerves were located up and down, and the mandibular marginal branch of the facial nerve was separated during the whole process. Then, the distal ends of the facial artery and vein were separated and disconnected along the surface of the submandibular gland, and the submandibular gland and the lymphoid adipose in region Ib were separated and resected (Fig. 7). Finding and exposing the marginal mandibular branches of the facial nerves at the intersections of the distal ends of the facial arteries and veins with the mandible: After the platysma flap was overturned, the distal ends of the facial artery and vein were found, and careful separation was conducted. The marginal mandibular branch of the facial nerve was found on the superficial surface of the facial artery and vein. The marginal mandibular branch of the facial nerve was dissected throughout by a two-way separation. The distal ends of the facial artery and vein were disconnected. The submandibular gland as well as the lymphoid and adipose tissues in region Ib were separated and excised (Fig. 8). Not exposing the marginal mandibular branches of the facial nerves, the superficial layer of the deep cervical fascia was used as the anatomical plane, and the surrounding tissues were carefully separated along the gland surface. During the process, the gland was slightly pulled down with devices to maintain a safe distance from the marginal mandibular branch and the submandibular gland body; moreover, the distal ends of the facial artery and vein were exposed, the facial artery and vein were disconnected close to the surface of the submandibular gland, blunt dissection was conducted along the circumference of the submandibular gland, and the lymphoid and adipose tissues in region Ib were separated and excised.

### Postoperative observation items

The anatomical position and state of the marginal mandibular branches of the facial nerves, as well as the function of the marginal mandibular branches of the facial nerves after surgery, were observed. After the operation, the function of the marginal mandibular branch of the facial nerve was investigated for 7 times until 1 year after the end of radiotherapy, namely, after surgery, before radiotherapy, after radiotherapy, 1 month after radiotherapy, 3 months after radiotherapy, 6 months after radiotherapy, and 12 months after radiotherapy.

### Statistical methods

The anatomical state and injury, as well as the occurrence and recovery of complications of the marginal mandibular branches of the facial nerves under the three different surgical procedures, were statistically analyzed. Chi-square test was conducted using SPSS 20.0, and the difference was considered statistically significant when *P* < 0.05.

## Results

### Selection of surgical procedures to protect the marginal mandibular branches of the facial nerves

Finding and exposing the marginal mandibular branches of the facial nerves at the mandibular angles of the platysma flaps was suitable for those with larger and more lymph nodes in region Ib. Finding and exposing the marginal mandibular branches of the facial nerves at the intersections of the distal ends of facial arteries and veins with the mandible was suitable for those with larger and more lymph nodes in region IIa. Not exposing the marginal mandibular branches of the facial nerves was suitable for those without obvious lymph nodes in region Ib or region IIa.

### The anatomical position of the marginal mandibular branches of the facial nerves

The marginal mandibular branches of the facial nerves penetrated from the front or lower ends of the parotid glands. They were constantly located between the deep surface of the platysma and the superficial layer of the deep fascia cervicalis and on the plane of the lower mandible. They crossed the superficial surface of the posterior facial veins, mandibular angles, and anterior facial veins from back to front, and entered the deep surface of deltoid muscles at the distal ends of facial arteries and veins.

### Different surgical procedures to deal with the marginal mandibular branches of the facial nerves

Among the 197 oral and oropharyngeal cancer patients undergoing comprehensive cervical lymphadenectomy with excision of the submandibular glands, the two methods for finding the marginal mandibular branches of the facial nerves both successfully found the marginal mandibular branches (Table 2).

**Table 2:** Different surgical procedures to deal with the marginal mandibular branches of the facial nerves (*n* = 197)

Parameter	Number of patients, *n* (%)
Finding and exposing the marginal mandibular branches of the facial nerves at the mandibular angles of the platysma flaps	102 (51.78)
Finding and exposing the marginal mandibular branches of facial nerves at the intersections of the distal ends of facial arteries and veins with the mandible	64 (32.49)
Not exposing the marginal mandibular branches of the facial nerves	31 (15.73)

### Injury of the marginal mandibular branches of the facial nerve

Among the 197 patients with oral and oropharyngeal cancer, four patients (4/197, 2.03%) had permanent injury of the marginal mandibular branches of the facial nerves. Among them, one patient (1/102, 0.98%) had an injury of the marginal mandibular branches of the facial nerves in whom the nerve was found and exposed at the mandibular angles of the platysma flaps; one patient (1/64, 1.56%) had an injury of the marginal mandibular branches of the facial nerves in whom the nerve was found and exposed at the intersections of the distal ends of the facial arteries and veins with the mandible; and two patients (2/31, 6.45%) had an injury to the nerve in whom the marginal mandibular branches of the facial nerves were not exposed.

The temporary injury occurred in seven patients (7/197, 3.55%). Among them, two patients (2/102, 1.96%) had an injury of the marginal mandibular branches of the facial nerves in whom the nerve was found and exposed at the mandibular angles of the platysma flaps; two patients (2/64, 3.13%) had an injury of the marginal mandibular branches of the facial nerves in whom the nerve was found and exposed at the intersections of the distal ends of the facial arteries and veins with the mandible; and three patients (3/31, 9.68%) had an injury to the nerve in whom the marginal mandibular branches of the facial nerves were not exposed.

There were statistically significant differences in the protection of the mandibular marginal branch of the facial nerve among the three different surgical methods (*χ*^2^ = 7.9875, *P* = 0.0184). The best protective effect was to find and expose the mandibular marginal branch of the facial nerve at the mandibular angle of the platysma muscle flap, and the injury rate was only 2.94% (Table 3). The patients were followed up for 6–90 months after surgery, and the functions were restored in all patients with temporary injury of the marginal mandibular branches of the facial nerves.

**Table 3:** Injury of the marginal mandibular branches of the facial nerve (*n* = 197)

Parameter	Number of patients, *n* (%)	No injury, *n* (%)	Injury n (%) Permanent injury n (%)	Temporary injury n (%)
Finding and exposing the marginal mandibular branches of the facial nerves at the mandibular angles of the platysma flaps	102 (51.78)	99 (97.06)	1 (0.98%)	2 (1.96%)
Finding and exposing the marginal mandibular branches of facial nerves at the intersections of the distal ends of facial arteries and veins with the mandible	64 (32.49)	61 (95.31)	1 (1.56%)	2 (3.13%)
Not exposing the marginal mandibular branches of the facial nerves	31 (15.73)	26 (83.87)	2 (6.45%)	3 (9.68%)
*P*				0.0184
*χ*^2^				7.9875

The difference was considered statistically significant when *P* < 0.05

## Discussion

With the development and progress of society, in the treatment of patients with locally advanced oral and oropharyngeal cancer, not only the survival rate but also the quality of life should be improved. The injury of the marginal mandibular branches of the facial nerves can cause dysfunctions, with a serious physiological and psychological impact on patients. Cervical lymph node metastasis is a characteristic of locally advanced oral and oropharyngeal cancer and an important factor for prognosis. Adequate assessment and lymphadenectomy are extremely important for improving the survival rate and quality of life in locally advanced oral and oropharyngeal cancer [15, 16].

Lymphadenectomy is the gold standard for surgical treatment of cervical lymph node metastasis. With the development of functional surgery, radical cervical lymphadenectomy characterized by the resection of internal jugular veins, sternocleidomastoid muscles, and accessory nerves has been used decreasingly [17, 18]. Under the concept of maximum dissection of cervical lymphoid and adipose tissues and maximum preservation of normal tissues, whether the internal jugular veins, sternocleidomastoid muscles, accessory nerves, and other tissue structures are spared does not affect the thoroughness of cervical lymphadenectomy, and selective lymphadenectomy and comprehensive lymphadenectomy are more in line with the concept of functional surgery [1]. Under the guidance of the concept of functional surgery, the preservation of submandibular glands in cervical lymphadenectomy has received increasing attention. There is enough evidence showing that it is safe and feasible to preserve the submandibular glands in cervical lymphadenectomy for early-stage oral and oropharyngeal cancer; however, it is still necessary to remove the submandibular glands in cervical lymphadenectomy for locally advanced oral and oropharyngeal cancer [19–21], and the removal of submandibular glands are closely related to marginal mandibular branches of the facial nerves [22].

The anatomy of the marginal mandibular branches of the facial nerves is complicated. The facial nerves run forward and downward from the stylomastoid foramina, pass through the posteromedial surface of parotid glands, and enter the parotid glands. The marginal mandibular branches are sent out from the cervical-facial trunks, pass through the front or lower end of parotid glands, pass through the mandibular angles, and are constantly located between the deep surface of platysma and the superficial layer of deep fascia cervicalis, and on the plane of the lower mandible. They cross the superficial surface of the posterior facial veins, mandibular angles, and anterior facial veins from back to front and enter the deep surface of the deltoid muscles at the distal ends of facial arteries and veins [23–25]. A marginal mandibular branch of the facial nerve is a fine nerve, which usually has 1–3 branches. After its injury, it leads to paralysis of the lower lip, bilateral facial asymmetry, significantly skewed mouth angle, salivation, and even impact on eating, which seriously affects the appearance and functions of patients, reduces the quality of life, and causes great psychological trauma to patients [9, 26]. How to protect the marginal mandibular branches of the facial nerves when resecting the submandibular glands in comprehensive cervical lymphadenectomy in patients with locally advanced oral and oropharyngeal cancer, whether to dissect them and how to dissect, is worth pondering.

In our multicenter retrospective study, according to the different characteristics of patients and cervical metastatic lymph nodes, as well as the proficiency of surgeons, we adopted three different surgical procedures to deal with the marginal mandibular branches of the facial nerves. For patients with larger and more lymph nodes in region Ib, the marginal mandibular branches of the facial nerves were found and exposed at the mandibular angles of the platysma flaps. For patients with larger and more lymph nodes in region IIa, the marginal mandibular branches of the facial nerves were found and exposed at the intersections of the distal ends of facial arteries and veins with the mandible. After finding the marginal mandibular branches of facial nerves in these two ways, the facial nerves were completely separated under direct vision, the proximal and distal ends of facial arteries and veins were disconnected, the submandibular glands were resected, and the lymphoid and adipose tissues in region Ib were cleaned. The dissection of the marginal mandibular branches of the facial nerves according to different characteristics of lymph nodes can avoid the inflammation and tissue adhesion caused by lymph nodes and protect the marginal mandibular branches of facial nerves to the greatest extent. For patients without obvious lymph nodes in region Ib or region IIa, the submandibular glands were removed, and the lymphoid and adipose tissues in region Ib were cleaned without exposing the marginal mandibular branches of the facial nerves, When cleaning the perivascular lymph nodes at the distal end of the facial artery and vein and ligating blood vessels to remove the submandibular gland, special attention should be paid to the position of the marginal mandibular branch of the facial nerve to avoid injury. However, there were statistically significant differences in the protection of the mandibular marginal branch of the facial nerve among three different surgical methods (*P* = 0.0184), suggesting that the mandibular marginal branch of the facial nerve should be dissected as far as possible in the comprehensive neck lymph node dissection for locally advanced oral oropharyngeal carcinoma. The best protection effect was to find and expose the mandibular marginal branch of the facial nerve at the mandibular angle of the platysma muscle flap, and the injury rate was only 2.94%.

Among the 197 patients with locally advanced oral and oropharyngeal cancer, the incidence of permanent injury of the marginal mandibular branches of the facial nerves was 2.03% and that of temporary injury was 3.55%. The extremely low incidence of injury of the marginal mandibular branches of the facial nerves verified our correct choices. We should conduct a sufficient evaluation before the operation, formulate a strict surgical plan, and flexibly choose the measures to protect the marginal mandibular branches of the facial nerves according to the specific situation of the primary lesion and lymph nodes as well as the surgeon’s proficiency. When the marginal mandibular branches of the sectional nerves are not dissected, the glands should be pulled down slightly with instruments to keep a safe distance from the marginal mandibular branches for resection. When dissecting the marginal mandibular branches of the facial nerves, we should pay attention to its branches [24, 25]. With thorough hemostasis, the nerves can be clearly and completely exposed during dissection. Electrocoagulation and clamping are prohibited in the peripheral areas of the nerves. The epineuria of the nerves should be protected, and exposure of the nerves should be avoided. Various risk factors that can cause injury to the marginal mandibular branches of the facial nerves should be minimized, thereby achieving the goal of maximum protection of the marginal mandibular branches of the facial nerves.

## Conclusion

The best protective effect was to find and expose the mandibular marginal branch of the facial nerve at the mandibular angle of the platysma muscle flap. Finding and exposing the marginal mandibular branches of the facial nerves at the mandibular angles of the platysma flaps was suitable for those with larger and more lymph nodes in region Ib. Finding and exposing the marginal mandibular branches of the facial nerves at the intersections of the distal ends of facial arteries and veins with the mandible was suitable for those with larger and more lymph nodes in region IIa. Not exposing the marginal mandibular branches of the facial nerves was suitable for those without obvious lymph nodes in region Ib or region IIa.

In the comprehensive treatment of locally advanced oral and oropharyngeal cancer, individualized and precise treatment is required, and every detail in the treatment should be finely managed. Protecting the marginal mandibular branches of the facial nerves from injury will play an increasingly important role in the comprehensive treatment of locally advanced oral and oropharyngeal cancer. Intraoperatively, we should choose an appropriate method in exposing the marginal mandibular branches of the facial nerves according to the different characteristics of patients and cervical metastatic lymph nodes as well as the proficiency of surgeons.

## Data Availability

Datasets are available on request from the corresponding author on reasonable request. The raw data and all related documents supporting the conclusions of this manuscript will be made available by the authors, without undue reservation, to any qualified researcher.
